# Assessment and Comparison of Molecular Subtyping and Characterization Methods for *Salmonella*

**DOI:** 10.3389/fmicb.2019.01591

**Published:** 2019-07-12

**Authors:** Silin Tang, Renato H. Orsi, Hao Luo, Chongtao Ge, Guangtao Zhang, Robert C. Baker, Abigail Stevenson, Martin Wiedmann

**Affiliations:** ^1^Mars Global Food Safety Center, Beijing, China; ^2^Department of Food Science, College of Agriculture and Life Sciences, Cornell University, Ithaca, NY, United States

**Keywords:** *Salmonella*, subtyping, serotyping, WGS, PFGE, MLST, food industry

## Abstract

The food industry is facing a major transition regarding methods for confirmation, characterization, and subtyping of *Salmonella*. Whole-genome sequencing (WGS) is rapidly becoming both the method of choice and the gold standard for *Salmonella* subtyping; however, routine use of WGS by the food industry is often not feasible due to cost constraints or the need for rapid results. To facilitate selection of subtyping methods by the food industry, we present: (i) a comparison between classical serotyping and selected widely used molecular-based subtyping methods including pulsed-field gel electrophoresis, multilocus sequence typing, and WGS (including WGS-based serovar prediction) and (ii) a scoring system to evaluate and compare *Salmonella* subtyping assays. This literature-based assessment supports the superior discriminatory power of WGS for source tracking and root cause elimination in food safety incident; however, circumstances in which use of other subtyping methods may be warranted were also identified. This review provides practical guidance for the food industry and presents a starting point for further comparative evaluation of *Salmonella* characterization and subtyping methods.

## Introduction

A number of food safety incidents and recalls caused by *Salmonella* contamination have been associated with ready-to-eat low-moisture products (e.g., milk powder, raw almonds, dry seasonings, and peanut butter) (Pillai and Ricke, 2002; Maciorowski et al., 2004; Park et al., 2008; GMA, 2009; Hanning et al., 2009), and other food commodities (e.g., meat products, eggs, and vegetables) (Greig and Ravel, 2009; Wu et al., 2017; Ricke et al., 2018) in recent years. These cases highlight the need to reinforce *Salmonella* control measures in the food industry, including rapid and accurate tracking of pathogen contamination sources with appropriate subtyping tools. Tools used in incident investigations that can differentiate *Salmonella* beyond the species level (defined as *Salmonella* subtyping) are essential to improve control of this pathogen, as *Salmonella* contamination can occur from diverse sources at any stage of food production (Olaimat and Holley, 2012; Barco et al., 2013; Shi et al., 2015).

Conventional serotyping (White–Kauffmann–Le minor scheme) has been used as a *Salmonella* subtyping method for >80 years (*Salmonella* Subcommittee of the Nomenclature Committee of the International Society for, Microbiology, 1934; Grimont and Weill, 2007; Guibourdenche et al., 2010; Dera-Tomaszewska, 2012; Shi et al., 2015) and has been a certified approach for public health monitoring of *Salmonella* infections for over 50 years (CDC, 2015). This method classifies the genus *Salmonella* into serovars (also known as “serotypes”) based on surface antigens including somatic (O), flagellar (H), and capsular (Vi) antigens (Brenner et al., 2000). More than 2,500 serovars of *Salmonella enterica*, the *Salmonella* species responsible for virtually all salmonellosis cases have been identified by conventional serotyping (Hadjinicolaou et al., 2009; Ferrari et al., 2017), but less than 100 serovars account for the vast majority of human infections (CDC, 2015). Due to the large variety of *Salmonella* serovars, a laboratory needs to maintain more than 250 different high-quality typing antisera and 350 different antigens for conventional serotyping of *Salmonella* (McQuiston et al., 2004; Fitzgerald et al., 2006). The turnaround time (i.e., time needed from isolate submission to a laboratory to receipt of the result) for serotyping a single isolate is usually >3 days. In some cases, it can take much longer (>12 days) as multiple antibody/agglutination reactions may be needed in a step-wise fashion to assign a final classification for complex serovars (Kim et al., 2006; Boxrud, 2010). Traditional serotyping is thus time-consuming and labor intensive requiring well-trained, experienced technicians (Boxrud, 2010; Shi et al., 2015). Unfortunately, it can also be imprecise (McQuiston et al., 2011). Moreover, the low discriminatory power of conventional serotyping may result in false-positive identification of relatedness between two unrelated isolates, as strains with the same serovar (such as the serovar *Salmonella* Enteritidis) may originate from multiple contamination sources. Further in-depth resolution beyond the serovar level is thus required for incident investigations (Ricke, 2017; Ricke et al., 2018). Various rapid molecular-based subtyping methods have been developed to provide faster, more discriminatory, and more accurate subtyping of *Salmonella* thus overcoming the limitations of traditional serotyping. Nevertheless, serovar data can still provide important historical epidemiological information, as certain serovars have specific virulence characteristics or may be associated with specific contamination sources (Ricke et al., 2018). Thus, it is important to link the subtypes identified by these molecular-based methods to *Salmonella* serovars.

There is no current global recommendation for the application of molecular characterization methods for *Salmonella*, although the food industry has applied both banding pattern-based and sequence-based subtyping methods for incident investigations. This review will provide (i) a comparison between classical serotyping and selected widely used molecular-based subtyping methods including pulsed-field gel electrophoresis (PFGE), multilocus sequence typing (MLST), and whole-genome sequencing (WGS, including WGS-based serovar prediction), and (ii) a scoring system to evaluate and compare *Salmonella* subtyping assays.

## Banding Pattern-Based and Sequencing-Based Characterization Methods for *Salmonell*a

There are two major types of molecular-based subtyping methods: (i) nucleotide banding pattern-based subtyping methods, representing the banding patterns generated from the restriction digestion or polymerase chain reaction (PCR) amplification of genomic or plasmid DNA (Wachsmuth et al., 1991; Hartmann and West, 1997) and (ii) sequencing-based subtyping, identifying variants at the single-nucleotide level of the selected gene markers or the entire genome of an isolate. A comparison of the resolution, turnaround time, ability of serovar prediction, cost, and feasibility of these methods is given below (Table 1).

**TABLE 1:** Overview of *Salmonella* characterization and subtyping methods.

**Method**	**Ability to identify or predict serovars**	**Ability to provide sensitive subtype discrimination**	**Time to results from a single colony**	**Commercial availability (time to results that can be expected from commercial labs)**	**Summary of value for food industry**	**Estimated reagent cost per isolate (instrument and labor cost not included)^1^**	**Service cost per isolate (provided by commercial labs)^1^**
Classical White–Kauffman serotyping	While *Salmonella* serovars are based on White–Kauffmann serotyping, serotyping does provide frequent misclassification (Petersen et al., 2002).	Very poor subtype discrimination; only valuable as subtyping method for rare and unusual serovars.	2–17 days (usually >5 days) (ECDC, 2015; Bopp et al., 2016)	2–4 weeks	Classical serotyping is likely to be replaced rapidly by WGS-based serovar prediction. Main value for industry is as a rapid confirmation and subtype screen if access exists to lab that can provide rapid turnaround time.	$5–65 (ECDC, 2015; Bopp et al., 2016)	∼$175
Pulsed-field gel electrophoresis (PFGE)	Intermediate ability to predict serovars	Good subtyping discrimination for most serovars. Some PFGE patterns are very common within some serovars (e.g., Pattern 4 for *Salmonella* Enteritidis)	4–6 days (ECDC, 2015; Bopp et al., 2016)	2–3 weeks	Has been the gold standard subtyping method for *Salmonella*, is likely to be replaced rapidly by WGS, starting with public health authorities and food regulators.	$7–50 (ECDC, 2015; Bopp et al., 2016)	$130–200
Multiple locus variable number of tandem repeats (VNTR) analysis (MLVA)	Intermediate ability to predict serovars	Good subtyping discrimination for most serovars. May perform better than PFGE for some serovars but worse for others.	1–2 days	NA^2^	Has been used as a secondary subtyping method to compensate the low discriminatory power of serotyping and PFGE for some *Salmonella* serovars; it is likely to be replaced rapidly by WGS, starting with public health authorities and food regulators.	$9–36 (Amirkhanian et al., 2006; Top et al., 2008; Schouls et al., 2009; ECDC, 2015)	NA^2^
Legacy multilocus sequence typing (legacy MLST)	Intermediate ability to predict serovars	Better than conventional serotyping and riboprinting, worse than PFGE and WGS.	1–2 days	2–3 days	Main value for industry is as a rapid confirmation and subtype screen, can be used to select the reference genome for WGS data analysis.	$30–82 (Ranieri et al., 2013; Shi et al., 2015)	∼$280
Whole-genome sequencing (WGS)	Currently available serovar-prediction software using WGS data work well for less common serovars. May not work for extremely rare serovars.	Best discrimination among molecular subtyping approaches	3–17 days (ECDC, 2015) (depends on sequencing capabilities. Usually 1 day after sequencing is finished).	2–8 weeks	For companies with high demand of isolates to be subtyped, WGS is probably the most affordable and fastest method that provides the best discrimination. In addition, *in silico* serotyping and *in silico* MLST can be done from the data to allow for comparison with historical isolates that have not been whole-genome-sequenced. Other information, such as presence of antibiotic resistance genes and virulence genes can be easily retrieved from the data. For companies with low demand, the costs of real-time sequencing may be prohibitive, requiring that old isolates must wait until more isolates are collected to be submitted together.	$60–230 (ECDC, 2015)	$100 (using Illumina HiSeq X series)–up to more than $500 (using Illumina MiSeq)

*^1^These cost estimates per isolate are based on (i) previous cost estimation reports and studies, (ii) official prices available on Internet, and/or (iii) personal communication with service providers and product vendors, as of June 2018, true costs may vary considerably based on number of isolates tested per run, labor costs, and region/country, etc. ^2^NA, not available.*

### Banding Pattern-Based Characterization Methods

#### Pulsed-Field Gel Electrophoresis (PFGE)

**FIGURE 1:**
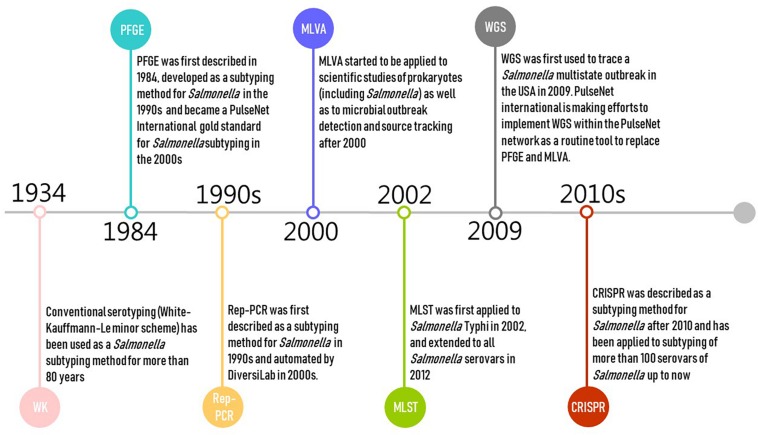
Timeline of the development of selected molecular subtyping and characterization methods for *Salmonella* (*Salmonella* Subcommittee of the Nomenclature Committee of the International Society for, Microbiology, 1934; Gilson et al., 1990; Threlfall and Frost, 1990; Hulton et al., 1991; Martin et al., 1992; Lindstedt et al., 2003, 2013; Healy et al., 2005; Grimont and Weill, 2007; Zou et al., 2010; Wattiau et al., 2011; PulseNet, 2014; CDC, 2016a, 2019; Nadon et al., 2017).

Pulsed-field gel electrophoresis was first described in 1984 and developed as a subtyping method for *Salmonella* in the 1990s (Threlfall and Frost, 1990; Figure 1). PFGE is currently the gold standard for PulseNet International, and has been used by public health authorities and food regulators for outbreak investigations and source tracking globally (including USCDC, USFDA, USDA, and ECDC) (Zou et al., 2010; Wattiau et al., 2011; PulseNet, 2014; CDC, 2016a). Alternative methods for *Salmonella* subtyping are commonly compared against PFGE (Call et al., 2008). However, PulseNet is transitioning from using PFGE and multiple locus variable number of tandem repeats analysis (MLVA) toward using WGS as the standardized genotyping method for foodborne pathogens (CDC, 2017a; Nadon et al., 2017). PulseNet International has defined standard PFGE protocols (PulseNet, 2013; CDC, 2017b) and maintains a database of *Salmonella* PFGE profiles with >350,000 PFGE patterns representing >500 serovars. These PFGE patterns predominantly represent isolates collected since 1996 in North America and Europe (Zou et al., 2013). PFGE has relatively high concordance with epidemiological relatedness with two decades of data accumulation (CDC, 2018a). However, the PulseNet database for PFGE patterns is not publicly available and can only be accessed by PulseNet participating laboratories.

The PFGE approach uses restriction enzymes that recognize specific restriction sites along the genomic DNA and fragment the DNA to sizes normally ranging from 20 to 800 kb (up to 2,000 kb) (Schwartz and Cantor, 1984; Singh et al., 2006). These large fragments are separated in a flat agarose gel by constantly changing the direction of the electric current (pulsed field), which causes the DNA to separate by size, generating a specific “fingerprint pattern” for a given isolate (Foley et al., 2009). The restriction enzymes *Xba*I, *Not*I, *Spe*I, and *Sfi*I have been typically used for Gram-negative bacteria including *Salmonella* (Barg and Goering, 1993). The primary restriction enzyme used for *Salmonella* PFGE is *Xba*I. A public health laboratory usually has access to software [e.g., BioNumerics and GelCompar (Applied Maths, Sint-Martens-Latem, Belgium); Diversity Database Fingerprinting Software (Bio-Rad Laboratories, Hercules, CA, United States)], to analyze a PFGE pattern (Nsofor, 2016) and uploads PFGE patterns to a national database. PulseNet Central’s database managers then analyze the uploaded pattern to see if a new outbreak has emerged or whether the isolate is part of an ongoing outbreak (CDC, 2018a). To make inter-laboratory comparison of DNA patterns possible, standardized protocols, molecular size standards (*Salmonella* Braenderup H2812, ATCC BAA-664), software, and nomenclature of PFGE patterns are required (PulseNet, 2015a). The approximate cost of the equipment and reagents required by PFGE can be accessed on the PulseNet International – PFGE site (PulseNet, 2015b).

Pulsed-field gel electrophoresis has been shown repeatedly to be more discriminatory than methods such as conventional serotyping, ribotyping, or MLST for many bacteria (Fakhr et al., 2005; Harbottle et al., 2006; Oloya et al., 2009; Soyer et al., 2010; Hauser et al., 2012). The combination of profiles generated by using additional restriction enzymes can enhance the value of this method for differentiating highly homogeneous *Salmonella* strains (Zheng et al., 2011); however, the cost increases as additional enzymes are used. PFGE can be used for subtyping of both Gram-positive (e.g., *Listeria monocytogenes*, *Staphylococcus aureus*) and Gram-negative (e.g., *Salmonella*, *Escherichia coli*, *Shigella*, *Campylobacter jejuni*) pathogenic bacteria. Typically, only the choice of the restriction enzyme and conditions for electrophoresis need to be optimized depending on the bacterial species investigated (PulseNet, 2015a).

Although various software platforms are available for PFGE pattern analysis, artifacts (e.g., brightly fluorescing spot) may lead to misidentification of bands. PFGE technology cannot usually be used to reliably visualize smaller fragments (e.g., <20.5 kb; Hunter et al., 2005) and has difficulty in differentiating bands differing by <5–10% in size due to the limited resolution of electrophoresis (Dijkshoorn et al., 2001; Persing et al., 2011). To address these issues, it has been recommended that users confirm PFGE pattern assignments using their experience and additional information to avoid incorrect band calling and systematic band shifts due to gel imperfections or imperfect reproducibility of electrophoretic conditions (Van Belkum et al., 2007). PFGE cannot be automated and requires high-level technical expertise and, thus, is hampered by low throughput, and may show low robustness and poor comparability of results between laboratories (Hyytia-Trees et al., 2007; Fabre et al., 2012; Kjeldsen et al., 2016).

No genetic information such as virulence potential or presence of antimicrobial resistance genes can be provided by PFGE, as the DNA fragments are separated by size rather than sequence (Ferrari et al., 2017). Observed bands of comparable size might not represent the same sequence of DNA, and a small mutation in a restriction site may result in changes in multiple bands. “Relatedness” determined by PFGE thus may not represent a true phylogenetic relationship between isolates (CDC, 2018a). Typically, multiple distinct PFGE patterns can be identified among isolates classified into the same serovar. Polyphyletic serovars, which are derived from more than one common evolutionary ancestor or ancestral group (e.g., serovars Newport, Mississippi, Saintpaul, Kentucky), show high levels of PFGE diversity (Porwollik et al., 2004; Sukhnanand et al., 2005; Alcaine et al., 2006; Harbottle et al., 2006; Sangal et al., 2010). PFGE-based prediction of these serovars is unreliable if isolates in the database are not representative of all clades of the serovar. On the other hand, PFGE may cluster epidemiologically unrelated isolates into identical PFGE types (Barco et al., 2013) and may even provide similar or identical PFGE types for isolates that represent different, but genetically very similar serovars that have a common ancestor (Barco et al., 2013; Shi et al., 2015), such as Typhimurium (antigenic formula: 1,4,[5],12:i:1,2) versus Typhimurium var. Copenhagen (antigenic formula: 1,4,12:i:1,2) (Heisig et al., 1995; Hauser et al., 2011), and Typhimurium versus 4,5,12:i:- (Guerra et al., 2000; Soyer et al., 2009; Wiedmann and Nightingale, 2009; Hoelzer et al., 2010; Ranieri et al., 2013). Furthermore, differentiation of genetically homogeneous serovars such as serovar Enteritidis challenges the usefulness of PFGE in *Salmonella* subtyping activities (Olson et al., 2007; Zheng et al., 2007). Approximately 45% of serovar Enteritidis isolates reported to PulseNet display the same PFGE *Xba*I pattern (JEGX01.0004), although many of these isolates are not epidemiologically related (Zheng et al., 2007). It is important to mention that the serovars mentioned above (i.e., Enteritidis, Typhimurium, Newport, Mississippi, Saintpaul, and Kentucky) are ranked among the most common *Salmonella* serovars associated with human and animal salmonellosis globally (Galanis et al., 2006; CDC, 2009).

#### Multiple Locus Variable Number of Tandem Repeats Analysis (MLVA)

Multiple locus variable number of tandem repeats analysis is a PCR-based typing method originating from forensic science where it is used for DNA “fingerprinting” samples of human origin. It has frequently been applied to scientific studies of prokaryotes as well as to microbial outbreak detection and source tracking (Lindstedt et al., 2003, 2013; Figure 1). MLVA is the second major genotyping tool (after PFGE) used in the PulseNet network (PulseNet, 2015c); prior to WGS, MLVA was one of the most popular subtyping methods used in public health surveillance and outbreak investigation of *Salmonella*, particularly in Europe (Torpdahl et al., 2007; Hopkins et al., 2011; Barco et al., 2013; Bauer et al., 2013; Lindstedt et al., 2013; Mughini-Gras et al., 2018). MLVA is usually performed following serotyping or PFGE for routine surveillance as a complementary technique for *Salmonella* subtyping (Torpdahl et al., 2007; Lienemann et al., 2015; Kjeldsen et al., 2016; CDC, 2017c; Ferrari et al., 2017), as it is challenging for PFGE to further differentiate isolates of genetically homogeneous serovars such as *Salmonella* Enteritidis (Kjeldsen et al., 2016). MVLA is especially used for typing *Salmonella* Typhimurium and *Salmonella* Enteritidis strains in reference or regulatory laboratories in Denmark, France, Germany, and United States [e.g., CDC, USDA – Food Safety and Inspection Service (FSIS) laboratories] (Barco et al., 2013; Bauer et al., 2013).

Multiple locus variable number of tandem repeats analysis is serovar specific, thus different *Salmonella* serovars usually require different MLVA schemes (Kruy et al., 2011). The first step toward uniform standardization of the MLVA profiles was collectively taken by PulseNet International and ECDC in defining the standard protocols of MLVA for *Salmonella* Typhimurium and *Salmonella* Enteritidis (ECDC, 2011, 2016b; PulseNet, 2015c). These serovars account for 26% of the culture-confirmed human *Salmonella* infections reported by US Laboratory-based Enteric Disease Surveillance (LEDS) and >60% of the salmonellosis cases reported by ECDC (ECDC, 2016a; Kjeldsen et al., 2016; CDC, 2018b). This uniform standardization of the MLVA profiles allowed direct comparison between laboratories irrespective of the platform used for MLVA (Larsson et al., 2009). Validated MLVA standard protocols for additional *Salmonella* serovars of clinical importance worldwide are largely missing, making MLVA use for serovars other than Enteritidis and Typhimurium difficult. However, with the advent of and transition into WGS, further development of MLVA may not occur.

Multiple locus variable number of tandem repeats analysis assesses the variation in the number of tandem repeated DNA sequences referred to as “variable-number tandem repeats” (VNTRs) in multiple regions of the bacterial genome to characterize bacterial isolates. The number of VNTRs in a given locus may vary between different microorganisms and even among bacterial isolates of the same species and serovar (Lindstedt et al., 2003; Torpdahl et al., 2007; Ngoi et al., 2015). The VNTR profiles vary in length between a few base pairs long to over 100 base pairs, enabling the development of techniques that utilize variation in the size of VNTR to discriminate closely related isolates (Lindstedt et al., 2003; Torpdahl et al., 2007; Fabre et al., 2012). The improved discriminatory power of MLVA varies with the serovar and phage type investigated (Torpdahl et al., 2007; Lienemann et al., 2015); e.g., in a study in Denmark, MLVA could differentiate distinct clusters within the most common phage types of *Salmonella* Typhimurium such as DT104, DT120, and DT12 even though these isolates displayed comparable PFGE patterns (Torpdahl et al., 2007). Public health laboratories usually have access to software (e.g., BioNumerics, GeneMapper, the free Peak Scanner) for analysis of MLVA patterns (ECDC, 2011; PulseNet, 2015c). Minimum spanning trees are frequently applied to MLVA profiles, yielding maps of predicted relationships among isolates based on single-locus and dual-locus variants (Van Belkum et al., 2007). However, web-accessible MLVA databases are not widely used for international collaboration (Guigon et al., 2008).

Multiple locus variable number of tandem repeats analysis is cheaper, faster, simpler to execute, and shows a relatively high-throughput compared with other molecular methods (Torpdahl et al., 2005, 2007; Lindstedt et al., 2007, 2013; Hopkins et al., 2011; Kruy et al., 2011). MLVA is less labor-intensive, time-consuming, and it is easier to perform than PFGE and MLST, as the protocol requires only a regular PCR step followed by capillary electrophoresis (Torpdahl et al., 2007; Lindstedt et al., 2013). Reduced handling time of pathogenic bacteria is beneficial for large-scale investigations. MLVA is also suitable for automation using a pipetting robot work station, automated sequencer, and analytical software (Barco et al., 2013; Lindstedt et al., 2013; Ferrari et al., 2017). Moreover, MLVA demonstrates good international repeatability and reproducibility for specific serovars such as *Salmonella* Typhimurium and *Salmonella* Enteritidis (Larsson et al., 2013). The data generated by MLVA can be readily analyzed and standardized for inter-laboratory comparisons (Torpdahl et al., 2007; Hopkins et al., 2011; Lindstedt et al., 2013; Wuyts et al., 2013).

A major drawback of MLVA for *Salmonella* subtyping is that the most effective MLVA protocols described so far are serovar-specific (Barco et al., 2013; Ngoi et al., 2015; Kjeldsen et al., 2016); hence, isolates have to be serotyped prior to selecting a specific MLVA scheme for further subtyping (Kjeldsen et al., 2016). At least 27 MLVA schemes have been developed to subtype different *Salmonella* serovars, whereas only *Salmonella* Typhimurium and *Salmonella* Enteritidis MLVA assays have been standardized in Europe and in the PulseNet network (PulseNet, 2015c; Kjeldsen et al., 2016). Another drawback is that rapid evolution of the target loci may decrease the reliability of results provided by MLVA regarding the relationship between strains under investigation (Hopkins et al., 2007, 2011; Lindstedt et al., 2013). This might hamper the use of MLVA, particularly in long-term epidemiological studies (Lindstedt, 2005; Li et al., 2009).

#### Repetitive Element PCR (Rep-PCR)

Repetitive element PCR targets the repetitive elements of genomic DNA to discriminate bacterial isolates. This method has been developed using three families of repeat sequences for subtyping *Salmonella*, including “enterobacterial repetitive intergenic consensus” (ERIC) sequences, “the repetitive extragenic palindromic” (REP) sequences, and the “BOX” sequences (Gilson et al., 1990; Hulton et al., 1991; Martin et al., 1992). The PCR products amplified from genome regions containing these repetitive elements are analyzed by agarose gel electrophoresis, and the banding patterns generated are used to investigate the genetic relatedness between bacterial isolates (Sabat et al., 2013). The DiversiLab system (bioMérieux, Marcy-l’Etoile, France) automated the whole process of the Rep-PCR subtyping approach after 2000 and has been used for subtyping pathogens in hospitals worldwide (Healy et al., 2005; Chenu et al., 2012; Sabat et al., 2013; Figure 1). As the low reproducibility of original Rep-PCR method may have resulted from variability in reagents and gel electrophoresis systems (Sabat et al., 2013), the application of the DiversiLab system with microfluidic capillary electrophoresis increased both the resolution and reproducibility of the Rep-PCR approach (Healy et al., 2005; Chenu et al., 2012; Sabat et al., 2013). However, the system has been discontinued, making Rep-PCR unavailable as a commercial platform.

The major advantages of this method include its relatively low cost (comparable to that of PFGE) and short turnaround time (within one day) (Sabat et al., 2013; Ngoi et al., 2015). However, the discriminatory power of Rep-PCR in subtyping *Salmonella* is reportedly lower than that of PFGE (Tiong et al., 2010; Thong and Ang, 2011; Elemfareji and Thong, 2013; Ngoi et al., 2015). Its relatively low reproducibility (which can at least be partially addressed by automation, such as in the DiversiLab system), and low accuracy of serovar prediction (Weigel et al., 2004; Wise et al., 2009) have limited its application in *Salmonella* subtyping.

### Sequencing-Based Characterization Methods

#### Legacy Multilocus Sequence Typing (Legacy MLST)

Multilocus sequence typing is a nucleotide sequence-based approach that assesses DNA sequence variations (i.e., allelic type) of typically three, four, or seven selected well-conserved, housekeeping genes, usually using Sanger sequencing technology (Liu, 2010; Achtman et al., 2012). Schemes targeting seven genes are typically considered the “classical” MLST approach; this typing approach was originally proposed for isolates of *Neisseria meningitidis* (Liu, 2010). In this review, we focus on the most widely used *Salmonella* scheme targeting seven housekeeping genes [*aroC*, *dnaN*, *hemD*, *hisD*, *thrA*, *sucA*, and *purE*; hereafter denoted as legacy MLST to distinguish newer approaches (described below)] (Li et al., 2009; Yun et al., 2015). It was first introduced for *Salmonella* Typhi in 2002 (Kidgell et al., 2002), and extended to all *Salmonella* serovars in 2012 (Achtman et al., 2012; Figure 1). Legacy MLST is mainly used in research studies, assessing the population genetics and evolution of *Salmonella*. Public Health England (PHE) started adopting the seven-gene MLST (based on WGS data) approach as a replacement for traditional serotyping in 2015 (Ashton et al., 2016).

Historical MLST data including legacy MLST sequence types are maintained on EnteroBase (Alikhan et al., 2018). As of November 2017, the number of legacy MLST sequence types for *Salmonella* has reached 3,929 (Alikhan et al., 2018). Legacy MLST analysis can be conducted online by entering the sequences of amplified genes. Allelic variation at each locus is cataloged and a sequence type is assigned by comparing the allele set. The strains are characterized by their unique sequence type. With the advent of next-generation-sequencing technologies, legacy MLST data can also be extracted directly from WGS data using bioinformatics pipelines (Achtman et al., 2012; Ashton et al., 2016). The relatedness of isolates subtyped by legacy MLST can be displayed as a dendrogram or a minimum spanning tree based on the matrix of pairwise differences between their allelic profiles (Francisco et al., 2009), or as a phylogenetic tree built directly from the nucleotide alignment of the seven genes.

Legacy MLST can deliver results more rapidly than PFGE (Shi et al., 2015; Yun et al., 2015; Table 1), and the publicly available databases and online query system enable legacy MLST results to be highly reproducible and exchangeable between laboratories. However, legacy MLST shows lower discriminatory power than PFGE and MLVA, which limits its application to further discriminate isolates within a given serovar (Torpdahl et al., 2005; Alcaine et al., 2006; Foley et al., 2006; Harbottle et al., 2006; Hauser et al., 2012; Ngoi et al., 2015), and for source attribution (Barco et al., 2013). Protocols targeting sequences in genes that change more rapidly than housekeeping genes have been developed to improve the discriminatory power of legacy MLST (Ross and Heuzenroeder, 2005, 2008).

#### Clustered Regularly Interspaced Short Palindromic Repeat-Based Subtyping (CRISPR-Based Subtyping)

The clustered regularly interspaced short palindromic repeat (CRISPR) typing method uses the diversity of the content of CRISPR loci to distinguish bacterial strains. The application of the CRISPR system for subtyping foodborne pathogens is discussed in detail elsewhere (Shariat and Dudley, 2014; Shi et al., 2015; Barrangou and Dudley, 2016; Ferrari et al., 2017; Ricke et al., 2018). Although the CRISPR system has been applied to the subtyping of at least 100 *Salmonella* serovars (Shariat and Dudley, 2014; Barrangou and Dudley, 2016), this approach is not widely used by public health authorities or food regulators (Ferrari et al., 2017).

Clustered regularly interspaced short palindromic repeat loci contain variable lengths of CRISPR spacers obtained from foreign nucleic acids of plasmids or bacteriophages (Shariat and Dudley, 2014; Wright et al., 2017). These CRISPR spacers are acquired or lost during evolution of the pathogen over time in a sequential manner (Ricke et al., 2018), thus constructing a unique set of DNA sequence patterns that may provide sufficient resolution for pathogen subtyping (Fricke et al., 2011; Barrangou and Horvath, 2012; Shariat and Dudley, 2014; Wright et al., 2017). For subtyping, amplified CRISPR loci PCR products are sequenced by Sanger sequencing technology (Liu et al., 2011). The CRISPR spacer sequences are analyzed to assign each locus with an allelic type. The combination of the allelic types of analyzed CRISPR loci determine the isolate’s allelic profile (also referred to as the isolate’s sequence type) and is used to investigate the relationships between isolates (Liu et al., 2011).

The CRISPR approach has been shown to be feasible for subtyping of *Salmonella* (Liu et al., 2011; Fabre et al., 2012; DiMarzio et al., 2013; Shariat et al., 2013a, b, c; Almeida et al., 2017). Liu et al. (2011) developed a CRISPR–multi-virulence-locus sequence typing (MVLST) approach using virulence genes *sseL* and *fimH* with CRISPR1 and CRISPR2 loci; this approach was used to compare 171 isolates representing nine serovars (Typhimurium, Enteritidis, Newport, Heidelberg, Javiana, I 4,[5],12:i:-, Montevideo, Muenchen, Saintpaul) and was reported to be able to subtype *Salmonella* with resolution at the outbreak level. CRISPR–MVLST using different schemes of virulence genes has also been applied by others for subtyping *Salmonella* (DiMarzio et al., 2013; Shariat et al., 2013a; Almeida et al., 2017). The results from these studies suggest that CRISPR–MVLST has a higher discriminatory power than legacy MLST (Ferrari et al., 2017); however, discrimination is lower than PFGE in some cases (Almeida et al., 2015). While CRISPR typing has a relatively short turnaround time (comparable to MLST), current major drawbacks include high cost (Almeida et al., 2017; Ferrari et al., 2017), unstandardized protocol, and database, as well as limited research on the concordance between the diversity of *Salmonella* isolates reflected by CRISPR loci content and by the other standard subtyping methods (Shi et al., 2015).

#### Whole-Genome Sequencing (WGS)

Whole-genome sequencing captures DNA sequence changes across the entire genome of single microbial isolates. The data are useful to assess evolution, allowing accurate description of the genetic relatedness of isolates. The use of WGS for *Salmonella* subtyping in outbreak investigation and pathogen source tracking has proven effective by a rapidly increasing number of studies (den Bakker et al., 2011, 2014; Allard et al., 2012; Leekitcharoenphon et al., 2014; Deng et al., 2015; Taylor et al., 2015; Hoffmann et al., 2016; Inns et al., 2016). WGS was first used to trace a *Salmonella* multistate outbreak in the United States in 2009 (CDC, 2019), and has been used for pathogen subtyping by the public health surveillance systems in the United States (Allard et al., 2018), Canada (Vincent et al., 2018), the United Kingdom (Ashton et al., 2016), Denmark (Kvistholm Jensen et al., 2016), and France (Moura et al., 2016). PulseNet international is also making efforts to implement WGS within the PulseNet network as a routine tool to replace PFGE and MLVA (Nadon et al., 2017; Figure 1). Both PHE (Ashton et al., 2016) and the US FDA (2018) have started using “real-time” WGS to subtype *Salmonella* isolates. CDC is also using WGS in state laboratories for *Salmonella* outbreak investigations (CDC, 2016b). WGS will be used increasingly for contamination incident investigations in the food industry, particularly as cost continues to shrink and ease of use increases. WGS (as well as other sequencing approaches that use the same next-generation sequencing technologies used for WGS) also have a number of additional applications in the food industry, which will further drive implementation of these tools. Examples of other applications include (i) monitoring ingredient supplies, (ii) identification of microbial persistence in processing environments, and (iii) prediction of antimicrobial resistance (including in *Salmonella*) and other relevant phenotypes, facilitating the improvement of sanitary management, microbial hazard control, and microbiological risk assessment (Allard et al., 2018; Rantsiou et al., 2018; Ricke et al., 2018).

Sequenced *Salmonella* genomes can be deposited and made publicly available on the National Center for Biotechnology Information site^1^, the European Bioinformatics Institute site^2^, or the DNA Data Bank of Japan site^3^ with data shared between all three (Kodama et al., 2012; Jagadeesan et al., 2019). NCBI provides phylogenetic tree-based clustering of all publicly available sequence data at the NCBI pathogen detection site^4^. These phylogenetic trees show the closest matches to any newly submitted data (Allard et al., 2018). NCBI also houses the data using GenomeTrakr Network (FDA, 2018). This was developed by the US FDA and NCBI as the first distributed network of laboratories to utilize WGS, with both genomic and geographic data, for foodborne pathogen characterization. This network includes the WGS laboratories of the CDC and USDA (Allard et al., 2016; Jackson et al., 2016). As of February 2019, there are over 184,000 genome sequences or raw sequencing data of *S. enterica* available on NCBI. WGS data analysis can also be performed off-line without using any public databases, an approach that may sometimes be preferred by industry.

Sequencing platforms that can be used currently for WGS include Illumina, Ion Torrent, Oxford Nanopore Technologies, and Pacific Biosciences (PacBio). Procedures to validate the complete workflow for *S. enterica* WGS with Illumina (MiSeq and HiSeq) and PacBio platforms from subculture of isolates to bioinformatics analysis have been reported by Portmann et al. (2018). The Illumina sequencing system is one of the most widely used sequencing platforms; it produces DNA-sequence reads with the length of 50–300 bp using sequencing-by-synthesis (SBS). This process uses fragmented DNA templates to detect single bases as they are incorporated during a DNA replication reaction on a solid surface flow cell (Illumina (2019)). For applications including comparative genomics and phylogeny, these short reads of DNA sequences can be aligned to a reference genome or *de novo* assembled into longer sequences called contigs (Loman and Pallen, 2015). The large amount of data generated by WGS combined with a complex data analysis process generally requires expertise in bioinformatics to deploy and run (Wyres et al., 2014; Deurenberg et al., 2017). Software with a more user-friendly interface, such as CLC Genomics Workbench^5^, BioNumerics, and Geneious (Biomatters, New Zealand), however, is available, including for industry users with limited bioinformatics expertise and an increasing number of user-friendly bioinformatics tools are being developed.

The rapid growth of WGS data in the publicly available databases allows industry to compare isolates with global entries of pathogen sequences used by food regulators and public health authorities (Allard et al., 2018; Rantsiou et al., 2018). Despite increasing availability of data analysis software, it is still challenging to generate consistent analytical reports due to the lack of standardized approaches to data analysis and interpretation (Clooney et al., 2016); for example, even with a standard software, choice of reference genomes can have considerable effects on the data analyses (Pightling et al., 2014). Furthermore, there are currently no clearly outlined safeguards to protect companies from regulatory action if shared WGS data show a relationship between pathogen isolates identified by a company and an outbreak isolate. Development of a mechanism for sharing data through anonymous hubs may allay concerns on confidentiality and encourage data sharing (FAO, 2016). This mechanism may also enable more effective data capture and analysis for monitoring trends and identifying related incidents.

The current cost of the entire WGS process, including DNA library preparation, sequencing, data analysis, and storage, is relatively high compared with the other molecular-based subtyping methods. The cost difference is more apparent when a small number of isolates are sequenced (as could be typical for the food industry). The cost of maintaining data analysis tools and bioinformatics personnel needs to be taken into consideration (Leekitcharoenphon et al., 2014; Ferrari et al., 2017; Nadon et al., 2017).

### WGS-Analysis Procedures

Interpretation of WGS data for source tracking or outbreak investigation typically uses two approaches to represent results: (i) single-nucleotide polymorphism (SNP) or allelic differences (often presented as distance matrix tables), and (ii) phylogeny or clustering of the isolates. SNP or allelic differences show objectively the genetic distance between two isolates. Hence, if isolate A shows three SNPs or allelic differences to isolate B, and 26 SNPs or allelic differences to isolate C, then we can say that isolate A is more similar to isolate B than to isolate C. If one assumes that all three isolates evolved at the same rate, then we can say that isolates A and B are evolutionarily more closely related to each other than they are to C. However, this assumption (i.e., all isolates evolve at the same rate) may not always be true. Environmental conditions or mutations in the DNA repair system may influence the rate of genetic change accumulated in a genome; e.g., a *Salmonella* isolate persisting in a humid, nutritious environment such as in a chicken farm may multiply much faster than an isolate persisting in a dry food processing environment. This environmental difference will allow the “chicken farm” isolate to accumulate more mutations (per year or any other time unit) than the dry food processing environment isolate, because the “chicken farm” isolate will multiply more times during the same period than the dry food processing environment isolate. Moreover, mutations in genes involved in DNA repair may result in the so-called “mutator phenotypes” (also sometime referred to as “hypermutators”). Mutator isolates accumulate mutations at a higher rate than non-mutator isolates (Muteeb and Sen, 2010). Hence, analyzing the number of SNP or allelic differences alone may result in misinterpretation of the results if the assumption that isolates evolved at the same rate does not hold true. Phylogenetic or clustering analyses are thus better suited to an investigation, as these analyses group isolates by their similarities instead of their differences (Pightling et al., 2018). To infer the evolutionary relationship of the isolates within a data set, therefore, a phylogeny must be constructed. For more detailed and technical information on reconstructing bacterial phylogenies from WGS data, the reader is referred to two in-depth reviews on this subject (Collins and Xavier, 2017; Patané et al., 2018).

#### WGS Analysis Approaches for Serotyping

Genetic-based approaches have been developed for *in silico* determination of serovars, because the phenotypic determination of *Salmonella* serovars is costly, time-consuming, and labor-intensive. These *in silico* methods have relied on two main approaches: (i) indirect determination using genetic markers associated with particular serovars and (ii) direct determination using genes responsible for the expression of the somatic O (*rfb* gene cluster) and flagellar H (*fljB* and *fliC*) antigens. The latter method has the advantage of relying on the same genetic information that results in the phenotype assessed by traditional serotyping, while the former method may require validation for new described serovars. These two approaches can also be combined for more reliable serovar prediction.

With the advent of whole-genome sequencing (WGS), *in silico* direct serovar determination has become the most used approach, and at least two *Salmonella* serovar databases and programs have been routinely used for *in silico* serotyping of *Salmonella*: SeqSero (Zhang et al., 2015) and SISTR (Yoshida et al., 2016a). SeqSero uses a database of 473 alleles representing 56 *fliC* antigenic types and 190 alleles representing 18 *fljB* antigenic types in a combined H-antigen database (Zhang et al., 2015). The somatic O-antigen database associated with SeqSero consists of 46 *rfb* gene cluster sequences corresponding to the 46 O-antigens identified in *Salmonella* (Zhang et al., 2015). The *rfb* database was specifically designed to be used with genome assemblies (as opposed to raw sequencing reads). A third database was specifically built for determination of the somatic O-antigen using raw sequencing reads (as opposed to genome assemblies). This third database consists of the genes *wzx* (encoding the O-antigen flipase), *wzy* (encoding the O-antigen polymerase), and other targets, all of which are found within the *rfb* gene cluster. In total, the authors claimed that the SeqSero scheme can theoretically identify 2,389 of the 2,577 serovars that were described in the White–Kauffmann–Le minor scheme by the end of 2014 (Zhang et al., 2015). The inability to predict 188 serovars is due to the absence of the DNA sequences for the antigen-encoding genes corresponding to these serovars in the SeqSero database. Empirical data showed that the SeqSero database has an accuracy of 91.5–92.6% for serotype prediction (Zhang et al., 2015).

SISTR is a platform for *in silico* analysis of *Salmonella* draft genome assemblies. SISTR includes the *Salmonella* Genoserotyping Array (SGSA) tool among other resources. SGSA relies on the allelic differences found within the *rfb* gene cluster for determination of 18 of the 46 somatic O-antigens, and *fljB* and *fliC* for determination of 41 flagellar H antigens (Yoshida et al., 2014). SGSA targets the identification of 90% (*n* = 2,190) of *Salmonella* serovars. When serovar determination using genoserotyping is not possible or is incomplete, SISTR also has the option to use the core genome MLST (cgMLST) scheme to infer the serovar based on phylogenetic context. The accuracy of SISTR in predicting *Salmonella* serovars has been assessed to be close to 95% (Yoshida et al., 2016a, b; Robertson et al., 2018).

Since SISTR can use genoserotyping and the cgMLST scheme to infer the serovar, higher confidence should be attributed to assignments where both genoserotyping and cgMLST agree on the serovar designation. Moderate confidence should be attributed to serovar assignments when only cgMLST is able to identify the serovar. When neither the genoserotyping nor cgMLST can identify the serovar, SeqSero may be used and may allow for serovar prediction.

### WGS Analyses for Subtype Characterization

#### Overview of WGS data analysis approaches

Different approaches can be used for analysis of WGS data for subtyping characterization related to source incident tracking. The most common approaches are based on (i) high-quality single-nucleotide polymorphism (hqSNP) identification and pairwise comparison of hqSNP differences, or (ii) whole-genome (wg)/cgMLST typing using pre-defined schemes (i.e., databases) containing allelic differences for either the pan (wg) or core (cg) genomes of *Salmonella* and subsequent pairwise comparison for assessing the number of allelic differences.

#### High-quality SNP analyses

High-quality SNP analyses rely on identification of SNP differences across a set of closely related isolates using raw sequence data, which are mapped to a closed or draft genome assembly (also referred to as the “reference genome”). Only SNPs that have been vertically transferred from an ancestral isolate to the current isolates are subject to the hqSNP analysis, while SNPs that were supposedly horizontally transferred are filtered out from the results. The reference can be a closely related genome outside the dataset, or a genome within the dataset. The analysis consists of two main steps: (i) mapping the raw sequence reads against the reference genome and (ii) SNP calling using stringent criteria to prevent the misidentification of sequencing errors or misaligned regions as SNPs (Davis et al., 2015; Katz et al., 2017). The choice of a closely related reference has been shown to be a key step in the analysis. Reference genomes that are not closely related to the set of isolates under investigation may result in underestimation of the number of SNPs, due to specific regions of the genome that may be present in the dataset under investigation, but that are missing in the reference genome (Pightling et al., 2014). There are at least two publicly available approaches that have been commonly used for hqSNP analysis: (i) the US FDA CFSAN (The Center for Food Safety and Applied Nutrition) SNP pipeline (Davis et al., 2015) and (ii) the US CDC-developed Lyve-SET hqSNP pipeline (Katz et al., 2017). These two pipelines rely on publicly available software to carry out the mapping and SNP calling steps and offer similar results despite some methodological differences, including different criteria for filtering out low-quality SNPs and masking regions supposedly acquired through horizontal gene transfer.

High-quality SNP analysis has been applied in several outbreak investigations in the United States, Canada, and some European countries, including a *Salmonella* Enteritidis outbreak in the United Kingdom that was linked to a German egg producer (Inns et al., 2015). Historical *Salmonella* Typhimurium isolates from humans and foods involved in five outbreaks and consisting of five distinct MLVA subtypes were re-analyzed using hqSNP analysis by Octavia et al. (2015); in this study at least 11 isolates not previously linked to the outbreaks were ruled in based on less than two SNP differences to the isolates previously linked to the outbreaks. Another retrospective study used hqSNP to analyze a collection of 55 *Salmonella* Enteritidis from seven epidemiologically characterized outbreaks and sporadic cases. One isolate not previously linked to any outbreak (i.e., sporadic) was identified to be part of one outbreak (“ruled in”) (Taylor et al., 2015). An investigation into a multi-state outbreak caused by *Salmonella* Poona was carried out in 2015 using PFGE and hqSNP analysis. Analysis by PFGE demonstrated three different patterns. However, WGS results showed that isolates with different PFGE patterns were genetically linked with less than six SNP differences (Kozyreva et al., 2016). Subtyping of *Salmonella* Dublin with PFGE was shown to have limited value in a recent outbreak investigation due to its low discriminatory power for this *Salmonella* serovar (Mohammed et al., 2016). The nine clinical isolates associated with the outbreak were indistinguishable by PFGE, but they were also indistinguishable from other unrelated *Salmonella* Dublin isolates. The nine isolates linked to the outbreak clustered together with one to nine SNP differences when analyzed using hqSNP, and they could be distinguished from other isolates that shared the same PFGE pattern with epidemiologically unrelated isolates showing more than 50 SNP differences when compared to the outbreak isolates (Mohammed et al., 2016). These studies show that public health agencies are increasingly relying on hqSNP analysis for outbreak investigation, including tracking the source of outbreaks. High-quality SNP analysis clearly improves subtype accuracy and outbreak investigations by not only allowing for increased discriminatory power, but also reducing instances where closely related isolates are being classified as “different.”

#### wgMLST

Whole-genome MLST (wgMLST) analysis relies on the comparison of individual genomes against a database containing all known alleles for all the genes representing the pan genome of a defined group of strains (i.e., serovar, subspecies, species, and genus). The pan genome is defined as all the genes present in at least one genome from a defined group. Two main approaches can be used, and these are often used in combination: (i) assembly free mapping and (ii) assembly based mapping. Raw sequencing reads are directly mapped against the database in an assembly free approach. Hence, this approach does not require *de novo* assembly of the genome prior to its utilization. SRST2 (Inouye et al., 2014) and BWA-MEM (Li, 2013) are the most commonly used programs to carry out this task. Because this approach deals directly with the raw sequence reads, it allows filtering low-quality reads or specific nucleotides with low quality within a good-quality read. In an assembly based approach the raw sequence reads are first used to generate a high-quality draft genome (i.e., usually not a closed genome) using a genome assembler. Later, the draft genome (i.e., assembly) is used to find matches against the database. The program most commonly used to map the draft genome against the database is BLASTN (Altschul et al., 1990), although other options also exist. Independently from the approach used (i.e., assembly free or assembly based), the result of mapping a genome against a database is a list of the alleles found in the analyzed genome. When more than one genome is analyzed, the list of alleles from each genome can be compared and the number of allele differences can be computed. Assembly free and assembly based wgMLST allele assignment should match for high confidence. Results are often shown as a distance matrix of allele differences and a dendrogram constructed from this distance matrix. The wgMLST methods allow for comparison of non-closely related isolates from different groups since all genomes are compared against the same database, which is a great advantage of this method over hqSNP (Maiden et al., 2013; Nadon et al., 2017). A disadvantage of the method is that the database must be constructed and shared across different groups, who must agree in using the same database in order to make their results comparable (Nadon et al., 2017). Construction of such databases is also time-consuming and labor-intensive, with the difficulty increasing with the diversity of the organisms included in the same database (e.g., a database for *S. enterica* subspecies *enterica* serovar Agona will require less time and labor than a database for all *S. enterica*).

#### Core genome MLST (cgMLST)

The cgMLST method is very similar to the wgMLST method. The major difference is the size and nature of the database. While the wgMLST database contains alleles for all genes in the pan genome of the defined group, the cgMLST only contains alleles for those genes that are present in all (or almost all) genomes of the defined group (i.e., the “core genome”). Hence, a cgMLST database will not capture the genetic diversity present in the accessory genes (i.e., genes that are not present in all isolates) and hence tends to be much smaller than a corresponding wgMLST database. The advantages of using the cgMLST are: (i) speed; because the cgMLST database is smaller than the wgMLST database, results can be obtained faster, and (ii) construction of the cgMLST database is generally easier than the wgMLST database, as typically less genomes are needed to identify the core genome than the pan genome of a group (den Bakker et al., 2010). While allele code schemes are used by some groups to summarize the differences observed among isolates subtyped by both cgMLST and wgMLST (Nadon et al., 2017), it generally is easier to define standard, stable, cgMLST allele codes. This allele code scheme can be easily transferred in a spreadsheet and can be interpreted similarly to what has been in use for PFGE. An allele code scheme may not, however, be fully stable and may need to be revised as new cg- or wgMLST types are identified (Nadon et al., 2017). A disadvantage of cgMLST is that it may show reduced discriminatory power over wgMLST, as shown in a comparison between the *Salmonella* cgMLST and wgMLST schemes defined in EnteroBase (Alikhan et al., 2018), carried out using *Salmonella* Enteritidis historical isolates from a UK egg-associated outbreak (Inns et al., 2015), as well as closely related non-outbreak isolates identified previously (Dallman et al., 2016). The 177 isolates from this dataset resulted in 177 unique sequence types by wgMLST (Simpson’s diversity index = 1.00) and 137 unique sequence types by cgMLST (Simpson’s diversity index = 0.98) (*P* < 0.05), showing the superior discriminatory power of wgMLST over cgMLST. However, both approaches grouped the isolates into identical clusters (Pearce et al., 2018).

#### Comparison of hqSNP-based analysis and genomic MLST analysis

Theoretically, hqSNP analysis is the most discriminatory approach for molecular subtyping, as it investigates all possible SNPs between each pair of isolates in the dataset. The second most discriminatory approach is wgMLST, which is designed to investigate virtually all genes in the genomes; intergenic regions and genes not present in the wgMLST scheme will not be investigated and polymorphisms present in these regions will be missed. The cgMLST approach is the least discriminatory of the three as it relies on only a subset of the genes present in the wgMLST scheme. Hence, similarly to the wgMLST approach, polymorphisms present in intergenic regions and in genes not included in the cgMLST scheme will not be assessed (Chen et al., 2017). Both wgMLST and cgMLST are reference-independent which makes the results more reproducible and transferable than hqSNP analysis (Nadon et al., 2017). In order to reproduce the results obtained from hqSNP analysis, one needs to use the same reference and parameters that were used in the original analysis (Nadon et al., 2017). This is not an issue with wgMLST or cgMLST analysis as long as analyses use the same scheme containing the same genes and alleles to allow for comparisons. Transference and communication of the results also seem to be more complicated for hqSNP analysis than for cgMLST or wgMLST (Nadon et al., 2017). This is because hqSNP analysis, as compared to cgMLST or wgMLST analyses, requires more parameter settings, which must be communicated for better interpretation. wgMLST and cgMLST analyses are also typically integrated into commercially available software, while the hqSNP pipelines are available as free open software or integrated into commercial software. Free-of-charge hqSNP pipelines require UNIX-based systems and are run through the command line, which may require specialized expertise (Nadon et al., 2017). Commercially available software, which can run cgMLST and wgMLST (e.g., BioNumerics) tends to be more user-friendly. BioNumerics uses a graphical user interface and can be installed in Microsoft Windows computers. The hqSNP analysis can easily be kept private as the analysis can be run within a closed dataset of genomes. The cgMLST and wgMLST can also be kept private; however, it may require some additional infrastructure (i.e., a private cloud) to be built around the commercial software.

### Comparison of Molecular Methods for Predicting the Serovar of *Salmonell*a

A comparison of different molecular methods for predicting the serovar of *Salmonella* is shown Table 2. Acceptable correlation between PFGE patterns and serovars has been described by several researchers (Weigel et al., 2004; Nde et al., 2006; Gaul et al., 2007; Kerouanton et al., 2007; Zou et al., 2010; Shi et al., 2015; Bopp et al., 2016). Shi et al. (2015) summarized the serovar-prediction accuracy of different molecular serotyping methods with studies from 1993 to 2013. The proportion of isolates that may not be accurately serotyped with PFGE is generally comparable to the proportion that is not typeable, or that requires extensive additional labor and reagents using conventional serotyping (Bopp et al., 2016). Examples of serovars incorrectly predicted by PFGE are summarized below (Table 3). Overall, with PFGE patterns for approx. 500 *Salmonella* serovars in the PFGE pattern database (Ranieri et al., 2013; Shi et al., 2015) and the reported good correlation between PFGE patterns and serovars, PFGE-based serovar prediction should be possible for a large proportion of these serovars, but will not be possible for a large number of less common serovars not represented in the database.

**TABLE 2:** Comparison of molecular characterization methods for prediction of *Salmonella*^1^ serovars.

**Number of isolates tested**	**Number of serovars tested**	**Isolate sources**	**Serovar-prediction accuracy (%)**	**References**
PFGE				
80	6	Turkey processing plant	99	Nde et al.,2006
68	10	Swine farms	84	Weigel et al.,2004
674	12	Swine	85	Gaul et al.,2007
866	8	Food animals, production facilities, and clinical samples	96	Zou et al.,2010
1,128	31	Food, animals, humans, natural environment, and processing plants	97	Kerouanton et al.,2007
46	40	Human and cattle	75	Ranieri et al.,2013
1,486	110	New York State Department of Health, isolates received in 2012; human clinics	96	Bopp et al.,2016
1,437	131	New York State Department of Health, isolates received in 2013; human clinics	91	Bopp et al.,2016
1,558	107	New York State Department of Health, isolates received in 2014; human clinics	90	Bopp et al.,2016
Legacy MLST				
25	7	Chickens	92	Liu,2010
66	1	Cattle, birds, horses, and other animals	99	Sukhnanand et al.,2005
110	25	Human and veterinary source	98	Torpdahl et al.,2005
152	33	Reference collection	100	Ben-Darif et al.,2010
4,257	554	Reference collection	88	Achtman et al.,2012
46	40	Human and cattle	91	Ranieri et al.,2013
42,400	624	SRA collection	91	Robertson et al.,2018
7,338	263	Human	96	Ashton et al.,2016
WGS-(SeqSero)				
308	72	CDC collection	99	Zhang et al.,2015
3,306	228	Genome Trakr collection	93	Zhang et al.,2015
354	44	GenBank collection	92	Zhang et al.,2015
WGS-(SISTR)				
4,291	246	SRA and NCBI Assembly collections	95	Yoshida et al.,2016a
42,400	624	SRA collection	97	Robertson et al.,2018

*^1^This table is revised from the information provided by the review of Shi et al. (2015).*

**TABLE 3:** Examples of serovars incorrectly predicted by PFGE.

**Major incorrectly predicted serovars**	**“O” antigens**	**Phase 1 “H” antigens**	**Phase 2 “H” antigens**	**References**
Montevideo (clustered with Senftenberg)	6,7	g,m,s	No phase 2 antigen	Nde et al.,2006
Senftenberg (clustered with Montevideo)	1,3,19	g,s,t	No phase 2 antigen	Nde et al.,2006
Typhimurium var. Copenhagen (clustered with 4,[5],12:i:- and Typhimurium)	1,4,12	I	1,2	Gaul et al.,2007
4,5,12:i:- (clustered with Typhimurium var. Copenhagen and Typhimurium)	4,5,12	I	No phase 2 antigen	Gaul et al.,2007
Typhimurium (clustered with Typhimurium var. Copenhagen and 4,[5],12:i:-)	1,4,5,12	I	1,2	Gaul et al.,2007
Saintpaul (clustered with Typhimurium var. Copenhagen and Typhimurium)	1,4,5,12	e,h	1,2	Ranieri et al.,2013
Putten (clustered with Agona)	13, 23	D	l, w	Gaul et al.,2007
Agona (clustered with Putten)	4,12	f,g,s	No phase 2 antigen	Gaul et al.,2007
Paratyphi B	1,4,5,12	B	1,2	Kerouanton et al.,2007
Give	3,10	l,v	1,7	Kerouanton et al.,2007
Newport	6,8	e,h	1,2	Kerouanton et al.,2007

Multiple locus variable number of tandem repeats analysis is not widely used for serovar prediction even though efforts have been made to develop MLVA subtyping schemes to subtype multiple serovars of *Salmonella* with one protocol (Van Cuyck et al., 2011; Kjeldsen et al., 2016). A universal MLVA scheme for most frequently isolated *Salmonella* serovars (accounting for 80% of the clinical isolates from humans in Europe) has been developed by Kjeldsen et al. (2016). In another study, an MLVA scheme identified 31 serovars (Van Cuyck et al., 2011). Nevertheless, further development of multiple-serovar MLVA schemes and robust MLVA profile databases is unlikely to occur given the benefits offered by WGS.

The serovar-prediction accuracy of Rep-PCR has been reported to range between 0 and 100%, indicating some limitations of this method (Shi et al., 2015). Ranieri et al. (2013) showed that Rep-PCR accurately predicted the serovar of 30 out of 46 isolates representing the top 40 *Salmonella* serovars isolated from human and non-human sources, with an accuracy of 65%. This accuracy was relatively lower than that obtained with PFGE or MLST, when the same set of isolates were evaluated.

Ashton et al. (2016) compared the serovars predicted by using legacy MLST sequences extracted from WGS data to the results generated by conventional serotyping, for 7,338 isolates representing 263 serovars of *Salmonella* enterica subspecies I. The 10 most common serovars in this *S. enterica* subspecies I dataset were serovars Enteritidis, Typhimurium, Infantis, Typhi, Newport, Virchow, Kentucky, Stanley, Paratyphi A, and Java. They found that the serovar prediction accuracy of legacy MLST was 96%.

The overall serovar-prediction accuracy for the CRISPR subtyping approach has been reported to range from 78 to 90% (Liu et al., 2011; Fabre et al., 2012; Shi et al., 2015). More studies are needed to further assess serovar-prediction accuracy using CRISPR.

Given the range of serovars represented in the SeqSero and SISTR databases, WGS can be used to theoretically predict 2,389 and 2,190 of the 2,577 serovars described in the White–Kauffmann–Le minor when using the serovar prediction programs SeqSero (Zhang et al., 2015) and SISTR (Yoshida et al., 2016a), respectively. Using empirical data, the accuracy of serotype prediction with SeqSero and SISTR has been reported to be approx. 92 and 95%, respectively (Zhang et al., 2015; Yoshida et al., 2016a, b; Robertson et al., 2018). By comparison, traditional *Salmonella* serotyping had an accuracy of 73% when 33–36 independent laboratories performed serotyping of the same eight *Salmonella* strains representing seven different serovars (Petersen et al., 2002), suggesting that WGS-based methods may be more reliable than traditional serotyping to assign *Salmonella* isolates to serovars. Nevertheless, further experimental studies are needed to continue to quantify the ability of WGS-based methods to identify *Salmonella* serovars.

### Comparison of Molecular Methods for Subtype Differentiation of *Salmonell*a

Molecular methods are used for subtyping *Salmonella* isolates that belong to the same serovar, as well as being used for serovar prediction. This section briefly provides some examples of comparative studies of subtyping methods. In one study, PFGE was compared to MLVA to subtype 163 non-typhoidal *Salmonella* isolates representing 15 serovars; MLVA differentiated the isolates into 79 MLVA subtypes while PFGE differentiated the same isolates into 87 subtypes. The Nei’s diversity index for MLVA was 0.979 compared to 0.999 for PFGE (Kjeldsen et al., 2016). However, for specific serovars (e.g., *Salmonella* Enteritidis) MLVA has been reported to provide improved discriminatory power over PFGE (Boxrud et al., 2007; Beranek et al., 2009; De Cesare et al., 2015). MLST has the advantage of being highly reproducible and easily transferable among laboratories. However, in a study of 110 *Salmonella* isolates from 25 serovars (Torpdahl et al., 2005), MLST resulted in 43 sequence types, while PFGE was able to differentiate the isolates into 73 PFGE subtypes. The downside of PFGE in this study was the inability to type 11 of the 110 (10%) isolates. In a study comparing different molecular methods to differentiate 52 *Salmonella* Enteritidis isolates, PFGE resulted in eight subtypes, while MLVA resulted in 18 subtypes and WGS resulted in 34 subtypes. The discriminatory power of PFGE, MLVA, and WGS was 0.81, 0.92, and 0.97 (Simpson’s index of diversity), respectively (Deng et al., 2015). In another study, PFGE and WGS were used to differentiate 55 *Salmonella* Enteritidis isolates; PFGE resulted in 10 subtypes; however, WGS was able to further differentiate the isolates into 45 unique subtypes (Taylor et al., 2015), showing the greater discriminatory power of WGS over PFGE. In a study of isolates from a *Salmonella* Poona outbreak (Kozyreva et al., 2016), 4 PFGE subtypes and 7 WGS subtypes were observed among the 16 isolates; *in silico* MLST using the WGS data resulted in one MLST sequence type. Phylogenetic analysis using WGS data showed that the distinct PFGE types did not necessarily correlate with increased genetic distance between isolates. Isolates that differed by 0 SNPs showed distinct PFGE subtypes, suggesting that PFGE results would be misleading for these isolates (Kozyreva et al., 2016). While the relative discriminatory power of different subtyping methods depends on the strains and serovars tested, WGS methods were consistently found to be most discriminatory, followed by PFGE. While some MLVA schemes provide enhanced discriminatory power over PFGE for some serovars, for other serovars PFGE may be more discriminatory than MLVA.

## Criteria to Evaluate and Validate Different *Salmonella* Characterization Methods

Molecular-based *Salmonella* characterization methods including WGS are evolving very fast. Many of the characterization methods and technologies, as well as data analysis pipelines, are operated as research tools, and are under continuous development. Evaluation of these tools for *Salmonella* investigation, especially for those serovars/strains highly relevant to food products and processing environments, is pre-requisite for the implementation of these methods. Methods that can be used by the food industry must be thoroughly validated before implementation to ensure reliability and consistency of the method when it is used across different laboratories. Validation should cover the end-to-end workflow for source tracking from isolate subculture to bioinformatic analysis, articulating the key quality requirements and criteria (Ferrari et al., 2017; Nadon et al., 2017; Portmann et al., 2018). Proposed criteria for evaluation of *Salmonella* characterization methods for potential routine use in the food industry are shown below (Table 4).

**TABLE 4:** Proposed evaluation criteria for *Salmonella* characterization methods that may be used routinely in the food industry^1^.

**Key criteria for evaluation**	**Description**	**Target**	**Key factors affecting performance**	**Quantitative evaluation (scale of 0–5)**
Stability	Consistency of the typing result for an isolate after its primary isolation and during laboratory storage and subculture.	Typing results should be stable during laboratory storage and subculture; strain markers should not mutate too rapidly to change the strain’s position in the epidemiological context; data on the stability of the markers should be available.	Rapid mutations and recombination of the marker(s) during storage and subculture could lead to poor reproducibility.	0 – Extremely poor stability 1 – No data are available on stability 3 – Some limited data suggest that markers are stable 5 – Strong data are available supporting stability of markers (and/or data are available that can be used to correct for mutations or changes in markers during passage).
Typeability	Ability to assign a type to all isolates tested by it.	Typeability should be as high as possible.	Poor typeability could be found in assays using a scheme that does not cover genetic variation in full; typeability may also be reduced if some isolates show high endogenous nuclease activity.	0 – Extremely poor typeability (<80%) 1 – Data indicate between 80 and 90% typeability; or no evaluation of typeability performed 2 – Data indicate between 90 and 93% typeability 3 – Data indicate between 94 and 96% typeability 4 – Data indicate between 97 and 99% typeability 5 – Data indicate >99% typeability.
Discriminatory power	Ability to assign a different type to two unrelated strains; discriminatory power can be expressed using Simpson’s index of diversity (SID)	Discriminatory power should be as high as possible. For highly discriminatory methods, clustering using phylogenetic analysis tools can be used to define isolates that share a recent common ancestor.	Discriminatory power is highly dependent on the marker(s) selected for typing.	0 – Extremely poor discriminatory power (<80%, SID <0.80) 1 – Data indicate between 80 and 90% discriminatory power (SID 0.80–0.90); or no evaluation of discriminatory power performed 2 – Data indicate between 90 and 93% discriminatory power (SID 0.90–0.93) 3 – Data indicate between 94 and 96% discriminatory power (SID 0.94–0.96) 4 – Data indicate between 97 and 99% discriminatory power (SID 0.97–0.99) 5 – Data indicate >99% discriminatory power (i.e., SID > 0.99). Note: we recommend that data are generated using appropriate strain collection and >100 isolates.
Epidemiological concordance	Ability to reflect, agree with, and possibly further illuminate the available epidemiological information about the cases under study.	Epidemiological concordance should be as high as possible; strains from the same outbreak or strains that are otherwise linked by epidemiological evidence should be classified into the same subtype (or phylogenetically characterized as sharing a recent common ancestor).	Low epidemiological concordance could be found in assays that either target “low stability markers” or an assay with limited discriminatory power, which will group together isolates that are epidemiologically unrelated.	0 – Extremely poor epidemiological concordance; <80% isolates are classified correctly. 1 – Poor epidemiological concordance; data indicate between 80 and 90% isolates are classified correctly; or no evaluation of epidemiological concordance 2 – Low epidemiological concordance; data indicate between 90 and 93% isolates are classified correctly 3 – Intermediate level of epidemiological concordance; data indicate between 94 and 96% isolates are classified correctly) 4 – Good epidemiological concordance; data indicate between 97 and 99% isolates are classified correctly 5 – Strong epidemiological concordance; data indicate all isolates are classified correctly Note: we recommend that data are generated by using at least 20 sets of epidemiologically related isolates. Ideally, a given subtyping method classifies all of these isolates correctly.
Reproducibility	Ability to perform reproducibly in different laboratories and with different personnel.	Results should be highly reproducible (>99%).	Poor reproducibility could be the results of (i) technically difficult assay (leading to technical errors by personnel, e.g., cross-contamination), (ii) reagents not standardized sufficiently, (iii) equipment not performing reproducibly, (iv) poorly optimized typing system, (v) sensitivity of equipment or assay system to environmental factors (e.g., humidity, temperature), (vi) bias in observing, recording, analysis, and interpretation of the results; (vii) or assays targeting biologically highly variable markers (e.g., some of the surface antigens targeted by classical serotyping).	0 – Extremely poor reproducibility; <80%; meaning for >20% of isolates results are not reproducible between labs 1 – Poor reproducibility; data indicate between 80 and 90% of isolates results are reproducible between labs 2 – Low reproducibility; data indicate between 91 and 93% of isolates results are reproducible between labs 3 – Intermediate reproducibility; data indicate between 94 and 96% of isolates results are reproducible between labs 4 – Good reproducibility; data indicate between 97 and 99% of isolates results are reproducible between labs 5 – Strong reproducibility; data indicate >99% of isolates results are reproducible between labs Note: we recommend that data are generated based on an evaluation by at least four laboratories.
Repeatability	Ability to produce the same results in the same laboratory with the same equipment and personnel	Results should be highly repeatable ( > 99%)	Poor repeatability could be the result of i) technically difficult assay (leading to technical errors by personnel, e.g., cross-contamination), ii) reagents not standardized sufficiently, iii) equipment not performing reproducibly.	0 – Extremely low repeatability (<90%; meaning for >10% of isolates results are not repeatable) 1 – No evaluation of repeatability performed 2 – Data indicate between 90 and 93% repeatability 3 – Data indicate between 94 and 96% repeatability; or repeatability evaluated with small number of isolates (<40) 4 – Data indicate between 97 and 99% repeatability 5 – Data indicate >99% repeatability Note: we recommend that repeatability evaluation performed with at least 40 isolates, ideally with 100 isolates.
Serovar prediction ability	Ability to accurately predict the serovar of a given strain.	Range, as the number of identifiable serovars, and accuracy (i.e., percentage of isolates with correct serovar identification) should be maximized. Accuracy should be given priority over range as misclassification may lead to worse decisions than non-classification.	Poor serovar prediction could be a result of (i) limited database coverage of different serovars, (ii) low discriminatory power, (iii) low typeability, (iv) no standard protocol of serovar prediction with produced data.	0 – Extremely low serovar prediction accuracy (serovar is correctly predicted for <70% of serovars) 1 – No evaluation of serovar prediction ability, or weak prediction accuracy (data indicate between 70 and 80% serovar prediction accuracy) 2 – Data indicate between 80 and 85% serovar prediction accuracy 3 – Data indicate between 85 and 90% serovar prediction accuracy; or serovar prediction ability evaluated with small number of serovars 4 – Data indicate between 90 and 98% serovar prediction accuracy 5 – Data indicate >98% serovar prediction accuracy); serovars are correctly predicted for all common isolates^2^ Note: we recommend that data are generated by using at least 40 different serovars, ideally more than 100 serovars.
Speed	Time to results from pure single colony	<5 days	Speed can be influenced by throughput, equipment, and data analysis program used for a given assay	0 – >1 month 1 – 3–4 weeks 2 – 2–3 weeks 3 – 1–2 weeks 4 – ≤5 days 5 – ≤2 days
Ease of use	Ease of use encompasses technical simplicity, workload, suitability for high throughput test, ease of data analysis, and result interpretation	Ease of use is important for the implementation of an assay in the internal laboratories of food industry, less important when using services provided by a commercial laboratory.	Poor ease of use is usually caused by the high level of expertise and experience required by a given assay, e.g., bioinformatics expertise to analyze data produced by the assay.	0 – The given assay requires extremely high level of expertise and experience in specific techniques (PhD level scientist with >4 days of specialized training) 3 – The given assay requires average level of expertise and experience of a microbiological technician 5 – No specific expertise or experience required; assay can be completed by high school diploma and <1 day training.
Cost	Total cost encompasses cost of equipment reagent/consumables, data analysis platform, and staffing. For routine use, we usually just assess the reagent cost per isolate. Staffing cost can vary considerably in different regions/countries within a given turnaround time, thus needs to be assessed separately with actual local situations.	A balance between efficiency/effectiveness and cost of a given assay is more important than pursuing low cost, because low cost may potentially lead to larger economic loss and extra investigation time caused by poor quality of typing result.	High cost per isolate for routine test is usually caused by high reagent cost and long turnaround time (leading to high staffing cost).	We recommend to use the actual reagent cost per isolate plus staffing cost estimated with given turnaround time to compare the assay being validated to the currently/previously used methods by food industry; data here are based on costs from commercial laboratories in North America and Europe: 0 – >$1,000 per isolate 1 – $500–$1,000 per isolate 2 – $200–$500 per isolate 3 – $150–200 per isolate 4 – $100–150 per isolate 5 – ≤$100

*^1^The parameters and information in this table are adapted from Van Belkum et al. (2007) and Wiedmann et al. (2014) with industry-specific practical needs. ^2^The serovar typing ability of conventional serotyping method (Kaufmann–White Le Minor scheme) is around 90% taking the typeability and accuracy of it into consideration (Bopp et al., 2016).*

## Implementation of Molecular-Based *Salmonella* Subtyping Methods by the Food Industry

We consider that WGS is the most suitable method to characterize *Salmonella* for incident investigation at production facilities in the food industry. This opinion is based on comparison of the resolution, turnaround time, ability of serovar prediction, cost, and feasibility of the available methods. Bioinformatics is a key capability required for WGS. The food industry may choose to invest in in-house capability that can interface with outside resources (e.g., academic partners, industry partners, government agencies), however, there are also opportunities to outsource data analyses to commercial or academic partner labs. Both the CFSAN pipeline and the Lyve-SET pipeline have been widely tested and seem to provide comparable and reliable results for hqSNP analysis. Implementation of wgMLST and cgMLST within BioNumerics has been successfully completed for *L. monocytogenes* in the United States. A cgMLST scheme is publicly available from EnteroBase (EnteroBase URL: https://enterobase.warwick.ac.uk/) and it is likely to be implemented within BioNumerics in the future. Other data analysis methods such as genome distance analysis (Pinho et al., 2009; Auch et al., 2010) can also become possible future approaches that allow for the food industry to develop data analysis capabilities for contamination source tracking.

The turnaround time of in-house WGS subtyping can be comparable to many conventional subtyping methods including conventional serotyping and PFGE (Table 1). WGS, however, provides much more information about an isolate with one single experimental procedure, enabling full characterization of the pathogen (including *in silico* serovar prediction and antimicrobial resistance gene identification) and more accurate clustering/discrimination of the isolates investigated. This is faster than using multiple conventional subtyping approaches in a stepwise approach to get equal information. The cost of WGS is also comparable to that of the conventional subtyping tools, considering the high quality and volume of information provided by WGS within one experimental procedure. *In silico* serotyping should be performed instead of traditional serotyping for determination of serovars once WGS is implemented as the subtyping method for *Salmonella*. This approach will greatly reduce the costs and time associated with serotyping.

Legacy MLST targeting variants of seven housekeeping genes of *Salmonella* can be used in combination with WGS. While legacy MLST classification can be obtained using Sanger sequencing technology (also known as first-generation sequencing technology) within 1 week, it can also be obtained by using the sequence information extracted from WGS data. Although legacy MLST has relatively lower discriminatory power compared with PFGE and MLVA, it is faster than PFGE when using an in-house Sanger sequencer such as Applied Biosystems Genetic Analysis Systems (Thermo Fisher Scientific). It is also more universal to all *Salmonella* serovars than MLVA which usually requires a specific scheme for each serovar. In addition, the serovar prediction ability of legacy MLST has been demonstrated to be comparable to that of PFGE (Tables 1, 3).

PFGE is currently still the “gold standard” and most widely used *Salmonella* DNA fingerprinting method used by public health authorities and food regulators to characterize and track this pathogen in outbreaks, although it is being replaced by WGS. PFGE remains a valuable tool for foodborne pathogen characterization by the food industry, while a transition to WGS occurs. PFGE has been repeatedly shown to be more discriminatory than methods such as conventional serotyping, automated ribotyping, or MLST for many bacteria including *Salmonella*. In addition to these methods, single-plex or multiplex PCR assays that can detect and identify specific *Salmonella* serotypes have been described (Kim et al., 2006; Akiba et al., 2011; Zhu et al., 2015; Xiong et al., 2018; Xu L. et al., 2018; Xu Y. et al., 2018); these tools provide an alternative approach for detection and identification of specific *Salmonella* serovars.

The results of any subtyping approach can be used to assess the relationship of isolates in an investigation. Nevertheless, the epidemiological context is indispensable in final decision making in incident investigation and to determine further actions for food safety management improvement. High-resolution WGS subtyping results should not be interpreted in the absence of epidemiological information.

The raw sequence data generated by molecular-based subtyping methods, especially WGS, require both physical and virtual space for storage. It is desirable to retain the original sequence reads (usually files with >200 MB for each *Salmonella* isolate) for potential future analysis using alternative data analysis methods or for a retrospective investigation. Commercial clouds can provide a storage solution, provided that special attention is paid to data security. A robust Internet connection and high band-width is needed to transfer WGS data if data storage is outsourced. Subtyping analysis needs to be supported by complete metadata providing the relevant epidemiological context to identify the root cause of the contamination. Thus, the capability for metadata collection, organization, and storage is needed together with building the capability for WGS. The metadata should include information such as the geographic and temporal background of the isolates, the sample type, and sample source (e.g., raw ingredients, finished products, environment), etc. The Consortium for Sequencing the Food Supply Chain, founded by IBM and Mars Incorporated, represents industrial groups putting effort into collecting genomic information on pathogenic bacteria across the food supply chain^6^. This consortium represents one part of the broader goal to increase knowledge of foodborne pathogens at the genomic level.

## Conclusion

The application of DNA-based methods for characterization of pathogens such as *Salmonella* has become common practice. Our literature-based assessment supports the superior discriminatory power of WGS and its advantages compared with other methods for *Salmonella* subtyping and source tracking for the food industry. We also identified circumstances under which use of other subtyping methods may be warranted. Implementation of molecular-based *Salmonella* characterization methods, including WGS, provides improvement of source tracking and root cause elimination; however, these methods require investment in bioinformatics capability. Routine use of WGS or complete replacement of current subtyping methods by WGS will require attention to key issues including standardization, robustness, and validation of the analytical methodology. High resolution WGS subtyping of *Salmonella* promises to vastly improve the ability of the food industry to track and control *Salmonella* and is poised to become standard methodology in food safety for characterization of foodborne pathogens by public health authorities and food regulators. Nevertheless, standardization of WGS operation and data analysis, in particularly source tracking analysis, is required at a global level. A common agreement of understanding and the application of WGS between the food industry, public health, and food safety regulators are expected to guide the implementation of WGS in food safety management.

## Author Contributions

ST and MW conceived and designed the work. ST, RO, and HL collected the data, conducted data analysis, and interpreted it. ST, RO, HL, and MW drafted the article. MW, AS, RB, CG, and GZ critically revised the article.

## Conflict of Interest Statement

ST, HL, CG, GZ, RB, and AS were employed by the Mars Global Food Safety Center. MW serves as a compensated scientific advisor for BioMérieux, Mérieux NutriSciences, Mars, and Neogen and has served as a paid speaker for 3M and IBM. The remaining author declares that the research was conducted in the absence of any commercial or financial relationships that could be construed as a potential conflict of interest.

## Footnotes

1 https://www.ncbi.nlm.nih.gov/sra

2 https://www.ebi.ac.uk/ena/

3 https://www.ddbj.nig.ac.jp/index-e.html

4 https://www.ncbi.nlm.nih.gov/pathogens/

5 https://www.qiagenbioinformatics.com

6 https://researcher.watson.ibm.com/researcher/view_group.php?id=9635
